# Research in tourism sustainability: A comprehensive bibliometric analysis from 1990 to 2022

**DOI:** 10.1016/j.heliyon.2023.e18874

**Published:** 2023-08-02

**Authors:** Chané de Bruyn, Foued Ben Said, Natanya Meyer, Mohammad Soliman

**Affiliations:** a Centre for Local Economic Development (CENLED), University of Johannesburg, South Africa; b University of Manouba, Tunisia; c DHET-NRF Sarchi Entrepreneurship Education, University of Johannesburg, South Africa; d University of Technology and Applied Sciences, Salalah, Oman; e Faculty of Tourism & Hotels, Fayoum University, Egypt

**Keywords:** Tourism, Sustainability, Tourism sustainability, Bibliometrics, Knowledge structures

## Abstract

Although multiple bibliometric studies have been conducted to analyze publications on various topics within tourism, little attention has been dedicated to systematically analyzing scholarly production on the topic of tourism sustainability. Consequently, this paper aims to conduct a comprehensive bibliometric and systematic review of tourism sustainability. The collected data include 6326 publications retrieved from the Scopus database. The bibliometric technique consists of two major analyses: one on the domain (levels of analysis) and one on knowledge structures. The results indicated a remarkable evolution of tourism sustainability research involving authors, sources, and publications on this subject. Several associations and nations made significant contributions to this theme. Moreover, science mapping approaches were used to thoroughly grasp tourism sustainability-related research's social, intellectual, and conceptual structure. By giving in-depth overviews and insights connected to tourism sustainability and its knowledge structures, this review article has various implications for scientific study and practice.

## 1 Introduction

The tourism industry is one of the world's fastest-growing industries. The industry is renowned for its potential to create employment opportunities, aiding in the fight against unemployment and poverty [[1], [2], [3]]. In 2019, before the COVID-19 pandemic weakened global economies, the tourism industry was responsible for 10.6% of global employment and 10.4% of the global GDP ([4]:3). Unfortunately, the devastating impact brought on by the pandemic due to strict travel restrictions being imposed across numerous countries worldwide, the industry's share in global GDP declined with over 49% ([4]:5). Nonetheless, as international restrictions continue to be eased, the industry is expected to recover substantially.

Even though sustainability within the tourism industry has started to attract more attention, researchers such as [5] and [6] express their concern regarding the slow adoption of sustainable practices within the industry. Moreover, the advancement in research relating to sustainability within the tourism industry has also received limited attention. Studies explicitly examining the progress and trends relating to research in sustainable tourism are limited. This could be problematic as, in an effort to ensure that research remains relevant and practical, a thorough understanding of current trends and shortcomings within the field is of the utmost importance.

Considering the abovementioned limitations, this study aims to provide a comprehensive overview of the evolution of research in sustainable tourism using bibliometric analysis. This study aims to answer three research questions, firstly, to identify the most fruitful authors, sources, and affiliations within the research domain. Secondly, to determine the intellectual structure by identifying and analyzing to most important co-citation clusters in tourism sustainability. Lastly, to design and develop a research agenda for future work on this important topic. It is worth mentioning that this study is unique in that it aims to provide a comprehensive bibliometric analysis of tourism sustainability published across all relevant journals from the start of the database Scopus (1990) till the end of December 2022, which sheds light on the evolution of the topic. Not only could this study contribute to the current body of research on sustainable tourism, but it could provide current and relevant guidance to researchers and policymakers in understanding the current trends and structure of research. Furthermore, it assists in identifying the main contributors and areas of research that could aid scholars in planning future research articles.

## 2 Literature review

As the world starts to recover from the effects of the global COVID-19 pandemic, the tourism industry could be one of the key economic sectors to kick-start economic recovery in especially developing countries. This view is supported by the known multiplier effect of the tourism industry, where the development of the industry not only benefits itself but also leads to spillover effects in other industries within the region ([7]:659; [8]:1). Despite the potential of the tourism industry to aid economic growth and development, a lack of appropriate and adequate policies and strategies could give way to negative externalities resulting from tourist activities. Research by [9] and [10] points out that if not properly managed, tourism could lead to biodiversity loss, pollution, and environmental degradation and negatively influence the social and cultural aspects of communities. Furthermore, researchers such as [11] and [2] express that the tourism industry is one of the world's leading contributors to greenhouse gas emissions. For that reason, sustainability is fundamental in tourism development.

Although various definitions of sustainability exist, it essentially suggests that development should not only meet the present needs but also aim to ensure that the needs of future generations are not compromised ([12]:39). Therefore, sustainable tourism refers to the ability of the industry to stay within the bounds of the region's carrying capacity, thereby not irreversibly disrupting the balance between the cultural, social and environmental features of the region ([13]:97). There is a growing concern for the need of more sustainable practices within the tourism industry [14]. For tourism businesses, the adaption of sustainable practices is not only crucial for their development and growth, but it also presents them with a competitive advantage ([10]:1). Burdett [15] describes the occurrence of unsustainable tourism, which refers to an increase in waste, air pollution, overconsumption of resources and ultimately suggest a type of tourism that does not support the livelihoods of the local community and environment.

Although the apparent importance of sustainability within the tourism industry has been highlighted, the industry has been criticized by scholars for its relatively slow adoption of sustainable practices [[5], [6]]. Although tourism research especially relating to its role in economies, is gaining more interest, sustainable tourism is still relatively new when compared to the research available on other economic industries. Buckley ([16]:537) writes that it is important to have a thorough knowledge of the various elements pertaining to the tourism industry, which includes the evaluation of research, as this allows not only academics but government policies to include more sustainable practices within the industry when they understand why and how the industry function. This is where bibliometric analysis proves useful.

Bibliometrics assesses the trend of research in various fields of study [17]. Ruhanen et al. ([18]:518) define bibliometric analysis as the quantitative, systematic review of academic literature in an effort to evaluate the scientific progress of the specific research field. Using bibliometric analysis allows researchers to explore research elements, trends in specific fields, intellectual structures of a research domain, journal and article performance and collaboration patterns [19]. Review studies and studies using bibliometrics within the tourism field have been gaining more popularity. Ye et al. [20] examined the various academic collaborations of a few journals relating to hospitality and tourism from 1991 to 2010 using bibliometric analyses. Shahbaz et al. [2] aimed to provide insight into tourism and its impact on environmental degradation using bibliometrics, analyzing articles from the Web of Science published between 1999 and 2020. Focusing on the sharing economy and tourism, Mody et al. [21] provided a critical review, using bibliometrics, of the research published in this field between 2010 and May 2020. Using bibliometrics, Wong et al. [22] focused on 12 journals listed in the Web of Science, where they examined the research activities of academics in the tourism and hospitality field. Providing insight post-COVID-19 into the research trends in tourism and hospitality, Wang et al. [23] analyzed articles from 18 Social Sciences Citation Index (SSCI) journals in Scopus using bibliometrics. In addition, Soliman et al. [24] systematically analyzed the main trends and themes in the leading tourism, leisure, and hospitality journals through an integrated bibliometric approach and network analysis before and during COVID-19. Analyzing literature relating to climate change and its impact on mountain tourism, Steiger et al. [25] conducted a systematic review analyzing 276 papers published within the Web of Science database.

With this being said, research using bibliometric analysis specifically relating to sustainable tourism is limited [[9], [18], [26]]. Drawing conclusions based on research trends in tourism and sustainability, Lu and Nepal [27] conducted a content analysis of all articles published between 1993 and 2007 in the *Journal of Sustainable Tourism*. Cavalcante et al. [10] used bibliometric methods to investigate the research published on the Web of Science pertaining to tourism and sustainability, specifically how it relates to marketing and branding. Looking at the development and leading trends in tourism sustainability and its relationship with employment and income, Garrigos-Simon et al. [26] used bibliometrics to analyze research published on the Web of Science up to December 2017. These authors suggest that the analysis be extended and that the use of other databases, such as Scopus, should be included in future studies.

Niñerola et al. [9] conducted a bibliometric analysis of sustainable tourism research published on Scopus up to 2018. Even though the study presents valuable insights, it only looked at the keywords appearing in the title, which limits the scope of the study. A study by Ruhanen et al. [18] aimed to identify trends in sustainable tourism research by employing a bibliometric analysis of research published in the four highest-ranked journals in the tourism field from 1987 through 2012. This study paves the way for future studies focusing on trends in sustainable tourism. However, focusing on only four journals does not provide a clear enough picture of the current trends as it has a narrow focus point. Another similar study by Moyle et al. [28] conducted a bibliometric analysis of sustainable tourism-related research published in top-ranked journals over a 30-year period. Their study indicates a definite need for improving theoretical and methodological research relating to sustainable tourism and its practicality. The authors point out that the limitation of this study lies in that it only focuses on the top-ranked journals, and certain influential studies from other journals could be excluded. In addition, the study only analyzed articles published up to 2017, and the search has been limited to keywords only, again limiting the reach of the methodology.

In light of the aforementioned limitations and areas of future interest highlighted in these similar studies, this study aims to provide a comprehensive overview of the evolution of research in sustainable tourism using bibliometric analysis.

## 3 Materials and method

### 3.1 Search strategies

In order to conduct a relevant bibliometric analysis of the tourism sustainability theme, a search of documents in the database of Scopus, the largest multidisciplinary database in the market, was adopted [29]. The search was performed according to the following criteria, articles published in English, including the theme ‘Tourism’ and ‘Sustainability’ in ‘titles, keywords and abstracts'. The search was carried out in March 2023. This has led to the elimination of articles published in 2023, as the year is incomplete. The search query is: (TITLE-ABS-KEY (tourism AND sustainability)) OR (TITLE-ABS-KEY (tourism AND sustainability AND cultur*)) OR (TITLE-ABS-KEY (tourism AND sustainability AND social*)) OR (TITLE-ABS-KEY (tourism AND sustainability AND economic*)) OR (TITLE-ABS-KEY (tourism AND sustainability AND environment*)) AND (LIMIT-TO (DOCTYPE, “ar”)) AND (LIMIT-TO (LANGUAGE, “English”)) AND (EXCLUDE (PUBYEAR, 2023)). This search query in Scopus retrieves articles that include the terms “tourism” and “sustainability” in their title, abstract, or keywords. The search also includes variations of these terms related to culture, social, economic, and environmental aspects. The results are limited to articles published in English. The document type is limited to research articles (ar).

### 3.2 Database

**Fig. 1:**
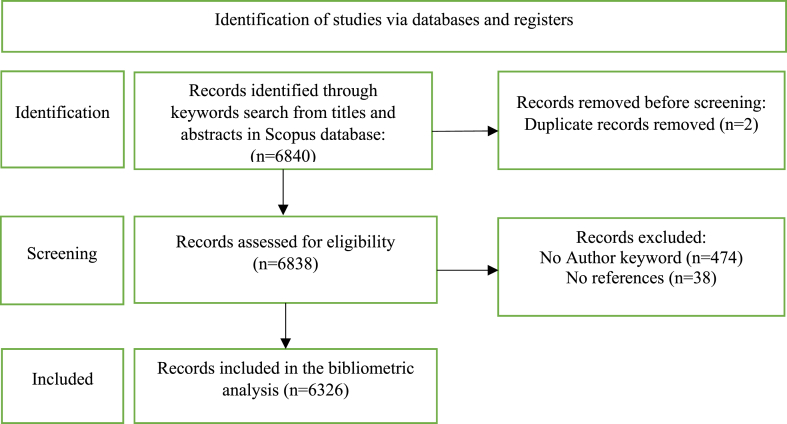
PRISMA diagram for inclusion of articles.

This search identified 6840 papers published in English journals. A systematic review of the downloaded database using the PRISMA 2020 method [30] resulted in the selection of 6326 eligible papers for the bibliometric analysis. Fig. 1 depicts the PRISMA diagram utilized for this study.

Table 1 contains the general information for this database. The papers were published between 1990 and 2022 in 1199 journals. The average age of a published paper is 6 years. A paper is cited 20 times on average. This database contains 342828 references and 14769 keywords. The number of authors who contribute to creating these documents equals 14347; 27% of the documents are written as an international collaborative effort of the authors.

**Table 1:** Main data information.

Description	Results
Timespan	1990: 2022
Sources (Journals, Books, etc.)	1199
Documents	6326
Annual Growth Rate %	21,62
Document Average Age	5,99
Average citations per doc	20,03
References	342828
DOCUMENT CONTENTS	
Keywords Plus (ID)	8330
Author's Keywords (DE)	14769
AUTHORS	
Authors	14347
Authors of single-authored docs	968
AUTHORS COLLABORATION	
Single-authored docs	1131
Co-Authors per Doc	3,01
International co-authorships %	26,97

### 3.3 Methodological approach

The bibliometric analysis of this database is divided into two parts. The first part included a descriptive analysis and was analyzed using the Bibliometrix package of the software R 4.2.2. It calculates annual production growth and identifies the most cited references or authors, local citations, and the most cited local authors. It calculates the authors' dominance ranking in the most productive sources and their h-index. In the analysis of co-citation and the construction of co-citation networks for authors, documents, sources and keywords, the method of [31] is used. This method automatically characterizes the clusters in terms of salient noun phrases extracted from the titles, abstracts and indexing terms of the cited articles and using representative phrases as summaries of the clusters. This procedure uses CiteSpace 6.2 R1 software and uses the following metrics to interpret networks and clusters:

a) Betweenness centrality: Determines at what level a specific node is in the path connecting two other nodes in the network [32]. Chen [33] indicates that high centrality values identify potentially groundbreaking scientific publications.
b) Silhouette: This metric was developed by Ref. [34] and measured the degree of uncertainty about the existence of a cluster. This metric varies between −1 and 1. A value close to 1 indicates that the cluster is perfectly separated from the others.
c) Burstiness: This metric allows testing for a frequency function, the existence of a significant fluctuation during a short period of time within a long period. It is useful to analyze co-citation networks to detect if and when the number of citations of a particular reference has increased [31].
d) Novelty or sigma: This metric noted by Σ allows us to identify the references that have constituted a novelty in a scientific field [35].
e) Cluster labels are selected from titles of cited papers, and the selection criteria are based on the log-likelihood ratios (LLR) ranking [36].

## 4 Results

The results constitute the analysis of the production of the documents as well as their quality through performance indicators. An analysis of the intellectual space is analyzed through the co-citation's networks. The subsequent section provides the various results.

### 4.1 Annual production

The annual scientific production curve shows the annual evolution of the scientific productions related to the tourism sustainability theme.

**Fig. 2:**
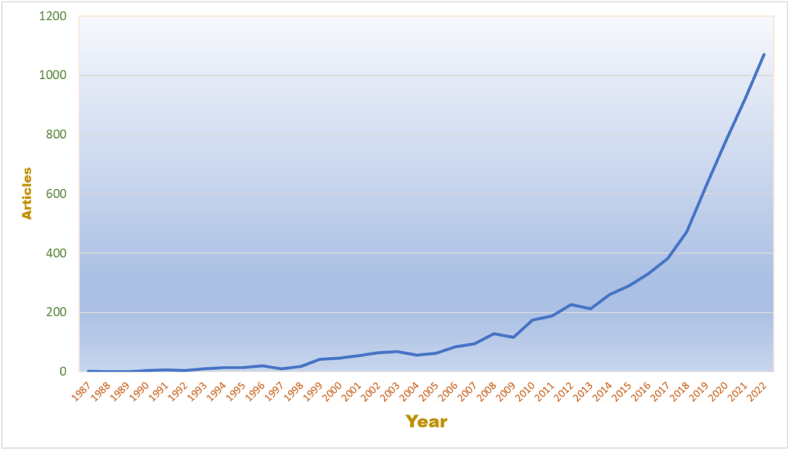
Annual scientific production.

Fig. 2 illustrates the evolution of the relevant published articles between 1990 and 2022. The theme has seen a sustained increase in production since 1998. Since 2014, the number of publications has exceeded 250 per year, and the growth rate will reach a thousand publications per year by 2022. The average annual growth rate is estimated at 21.62 articles per year.

### 4.2 Sources productivity

In this section, we evaluate the journals dealing with the tourism sustainability theme. The purpose is to identify the core sources of sustainability tourism in order to help researchers choose a suitable journal to publish their research [37]. Table 2 classifies the journals according to the h-index, a ranking metric. According to Ref. [29], the h-index combines the assessment of both the number of papers (quantity) and the impact or citations to these papers (quality). Therefore a source with an h index has at least h documents with h citations [29].

**Table 2:** 10 Most productive sources classified by h-index.

Element	h_index	TC	NP	PY_start
Journal of Sustainable Tourism	76	19862	455	2004
Tourism Management	65	12502	120	1996
Sustainability (Switzerland)	45	11977	907	2013
Annals of Tourism Research	38	6029	64	1994
Current Issues in Tourism	31	2803	94	2003
Tourism Geographies	30	3067	68	1999
Journal of Cleaner Production	29	3485	76	2005
Tourism Management Perspectives	26	1570	56	2012
Journal of Travel Research	24	1645	38	2004
International Journal of Tourism Research	22	1225	46	2008

**Note: TC:** Total Citations**; NP:** Number of Publications**; PY:** Publication Year.

The *Journal of Sustainable Tourism* occupies the first place in terms of h-index (76) due to the high number of citations of its papers. The most productive journal is *Sustainability*, with a total of 907 papers and an h-index of 45. The journal *Tourism Management* is ranked second in terms of the h-index. Annals of Tourism Research is the oldest journal in the publication of papers related to sustainable tourism and occupies the fourth place with an h-index equal to 38.

**Fig. 3:**
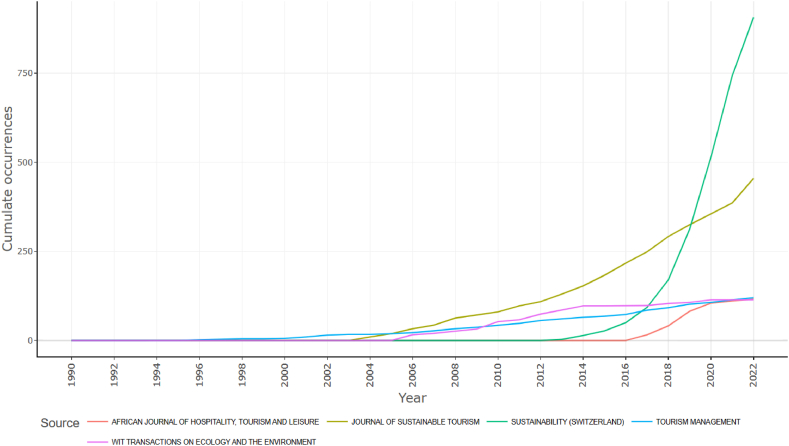
Sources dynamics.

The analysis of the evolution of production by source (Fig. 3) shows that the journal Sustainability (Switzerland) had the highest number of publications. Yet, it only started publishing papers related to tourism sustainability in 2013. The Journal of Sustainable Tourism comes in second place with 907 papers. In 2017 the African Journal of Tourism, Hospitality and Leisure started to publish papers related to this theme. In 2022 it occupied fourth place with 116 published papers.

### 4.3 Authors' production and citation

This section analyses the authors' classification using the dominance and h-index factors. The dominance factor that Kumar and Kumar (2008) developed refers to the ratio dividing the multi-authored publications in which the author appears first by the total number of publications. In the case where the author is the first author in all his multi-authored papers, then the DF = 1.

**Fig. 4:**
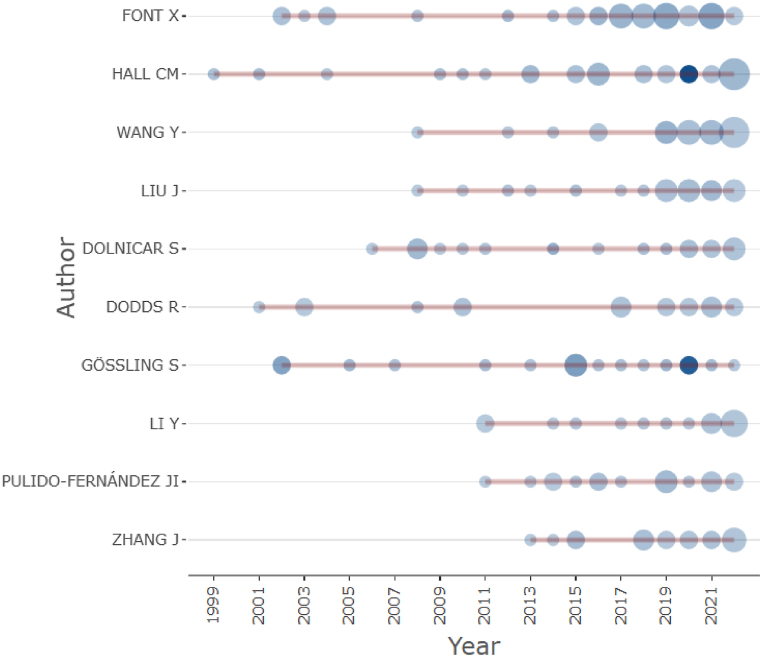
10 most productive authors over time.

Fig. 4 shows that Xavier Font started production in 2001. The year 2019 has recorded the largest publication of documents, with 6 articles on the theme. The author C. M. Hall started publishing papers with the theme of tourism sustainability in 1999 and reached the peak of his production in 2022 with 11 documents.

Looking at authors dominating the research field regarding tourism sustainability, Table 3 shows that Jamal T has the highest dominance factor of 0.6. This suggests that this author appears first in 60% of these publications. The second most dominant researcher is Gössling S, with a dominance factor of 0.583, having fewer single-authored articles than Jamal T.

**Table 3:** Author's dominance classification.

Author	Dominance Factor	Total Articles	Single-Authored	Multi-Authored	First Authored	Rank by Articles	Rank by DF
Jamal T	0,600	16	1	15	9	8	1
Gössling S	0,583	18	6	12	7	6	2
Dodds R	0,533	16	1	15	8	8	3
Weaver D	0,455	19	8	11	5	5	4
Dolnicar S	0,429	17	3	14	6	7	5
Font X	0,353	35	1	34	12	1	6
Hall C	0,313	24	8	16	5	2	7
Liu J	0,238	21	0	21	5	4	8
Wang Y	0,227	22	0	22	5	3	9
Kim S	0,214	16	2	14	3	8	10

Table 4 presents the authors' ranking according to their h-index. The author Xavier Font has the highest h-index (23) due to the high number of publications (39) combined with the number of citations which amounts to 1928. His paper entitled “Environmental certification in tourism and hospitality: progress, process and prospects” [38] is the most cited, with 632 citations. The author C. M. Hall occupies the second place in terms of h-index (19), and his most cited paper related to Tourism Sustainability is “Trends in ocean and coastal tourism: the end of the last frontier?” [39]. Stephan Gössling is the third author in terms of the h-index and has the highest total number of citations (3482). His paper entitled “Global environmental consequences of tourism” [40] is the most cited, with 796 citations. This paper investigates the environmental effects of international tourism by considering five major dimensions: land use change, energy use and related impacts, biota exchange and species extinction, disease exchange and dispersal, and psychological impacts on the environmental perception and comprehension caused by travel.

**Table 4:** Authors’ classification by h-index.

Element	h_index	TC	NP	PY_start
FONT X	23	1928	39	2002
HALL CM	19	2463	33	1999
GÖSSLING S	17	3482	18	2002
JAMAL T	14	919	15	2005
DOLNICAR S	13	1142	19	2006
BECKEN S	12	899	15	2001
HIGGINS-DESBIOLLES F	12	689	14	2008
RUHANEN L	12	472	15	2004
SCOTT D	12	1491	15	2006
WALL G	12	523	14	2009

### 4.4 Cited references analysis

Table 5 presents the articles that received the highest number of citations. The most cited paper is by Lui Z (2003) [41]. The importance of this paper lies in that it demonstrates the shortcomings in the literature by presenting the six approaches dealt with in this literature. The article with the second most citations is that of Buckley R (2012) [42]. This article aimed to assess the progress made relating to research in sustainable tourism by evaluating the focus, scope and results of publications concerning sustainable practices implemented by tourism businesses.

**Table 5:** Top 10 most cited documents.

Publication	Citations
Liu, Z. (2003). Sustainable tourism development: A critique. *Journal of Sustainable Tourism*, 11 (6):459-475	[43]
Buckley, R. (2012). Sustainable tourism: Research and reality. *Annals of Tourism Research*, 39 (2):528-546	[44]
Sharpley, R. (2000). Tourism and sustainable development: Exploring the theoretical divide. *Journal of Sustainable Tourism*, 8 (1):1-19	[45]
Hunter, C. (1997). Sustainable tourism as an adaptive paradigm. *Annals of Tourism Research*, 24 (4):850-867	[45]
Saarinen, J. (2006). Traditions of sustainability in tourism studies. *Annals of Tourism Research*, 33 (4):1121-1140	[46]
Bramwell, B. & Lane, B. (1993). Sustainable tourism: An evolving global approach. *Journal of Sustainable Tourism*, 1 (1):1-5	[47]
Braun, V. & Clarke, V. (2006). Using thematic analysis in psychology. *Qualitative Research in Psychology*, 3 (2):77-101	[48]
Butler, R.W. (1999). Sustainable tourism: A state-of-the-art review. *Tourism Geographies*, 1 (1):7-25	[49]
Miller, G. (2001). The development of indicators for sustainable tourism: Results of a Delphi survey of tourism researchers. *Tourism Management*, 22 (4):351-362	[50]
Choi, H.C. & Sirakaya, E. (2006). Sustainability indicators for managing community tourism. *Tourism Management*, 27 (6):1274-1289	[51]

### 4.5 Key words analysis

The distribution of the keywords according to the number of appearances is presented in Table 6. The most used keyword is *“sustainability”,* with 2456 citations. The median year of use of this word is 2018, indicating that 50% of the times of the appearance of this word has been realized in the last four years. The word *“ecotourism”* occupies the second place with 1252 uses, of which 50% have been realized in the last four years. The keywords *“sustainable tourism”* and *“environment”* achieved the 50% mark two years earlier in 2016.

**Table 6:** Top 20 most cited keywords.

Item	freq	year_q1	year_med	year_q3
Sustainability	2456	2014	2018	2021
Ecotourism	1252	2013	2018	2020
Sustainable development	1162	2014	2018	2021
Tourism	892	2017	2020	2021
Tourist destination	651	2015	2019	2021
Tourism management	580	2011	2017	2020
Tourism market	322	2014	2019	2021
Perception	278	2017	2019	2021
Tourism economics	253	2012	2017	2020
Environmental protection	211	2011	2016	2020
Environmental impact	190	2012	2016	2020
Economic development	183	2016	2020	2021
Biodiversity	167	2011	2017	2020
Protected area	150	2012	2016	2020
Covid-19	116	2021	2022	2022
Conservation management	101	2012	2015	2020
Water supply	91	2010	2015	2020
Carbon dioxide	79	2019	2021	2022
Nature conservation	73	2010	2015	2019
Land use	71	2009	2013	2019

Table 6 indicates that Sustainability is the guiding concept of sustainable tourism. Ecotourism is a subset of sustainable tourism focusing on nature-based tourism activities emphasizing conservation and education. Sustainable development is a key component of sustainable tourism, balancing economic growth with environmental protection and social well-being. Tourism management aims to optimize the benefits of tourism while minimizing its negative impacts. A sustainable tourism market requires awareness of environmental and social responsibility. Positive perceptions of the impacts of tourism can lead to increased tourism demand and support for sustainable tourism practices. Environmental protection and conservation are key sustainable tourism goals, including protecting and promoting biodiversity. COVID-19 has highlighted the importance of sustainable tourism practices and the need to balance economic growth with environmental protection and social well-being while ensuring resilient tourism models.

### 4.6 Intellectual structure

The intellectual structure uses science mapping tools such as the networks of co-citation of papers, sources and authors. The number of citations determines the most influential document, sources and authors and enables the most influential themes in a research field to be ascertained [52].

#### 4.6.1 Papers co-citation

**Fig. 5:**
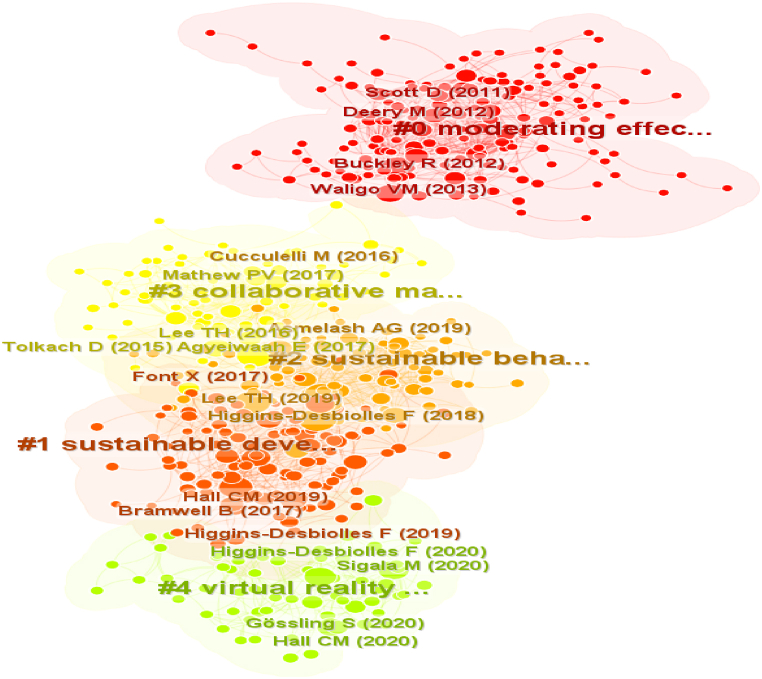
Network of largest 5 clusters of co-citation papers.

Documents are co-cited if they co-occur within the references of a third document. This analysis reveals the clusters formed by connecting documents following the same theme. The clusters' labels representing the dominant theme are calculated using the maximum likelihood ratio (LLR) statistical technique. Fig. 5 illustrates the five largest clusters in the documents cocitation network.

The largest cluster illustrated in red, labeled *sustainable tourism* by LLR, has 162 members and a silhouette value of 0.846. The silhouette score indicates cluster homogeneity, whereas silhouette scores close to 1 indicate that the cluster is more homogeneous ([53]. The leading citing article in this cluster is that of [54], titled Assessing tourism's global environmental impact 1900–2050. In this study, the author measures the global resource use and environmental impacts of tourism, introducing the concept of resource use intensity (RUI) to yield a preliminary estimate of the resource requirements of tourism per unit of consumption. The most cited papers in this cluster are the documents of Buckley [42], which discuss the environmental and social implications of the established tourism sector worldwide. The second most cited paper is Waligo et al. (2013), that discuss stakeholder involvement in sustainable tourism and reports on a UK-based case study that assessed stakeholder involvement in sustainable tourism management.

The second largest cluster has 114 members, a silhouette value of 0.724, and is labeled as *sustainable development* by LLR. The major citing article here is Molina-Collado et al. [55], titled *Sustainability in hospitality and tourism: a review of key research topics from 1994 to 2020,* aimed to analyze scientific research on sustainability in hospitality and tourism from 1994 to 2020 using bibliometric analyses and scientific mapping and to discuss implications for future research opportunities. Another most cited document is that of C.M. Hall [56], with 41 citations. Despite lacking a specific tourism section in the 2030 Agenda, this article explores how the UN's SDGs influence the policies and practices related to the sector. It argues that a more reflective approach is needed to understand the management and knowledge related to the sector. The second most cited document is by Higgins-Desbiolles et al. [57], which argues that the increasing interest in degrowth is a response to the harmful effects of neoliberal capitalism.

The third largest cluster in dark green has 89 members and a silhouette value of 0.827. It is labeled as *sustainable behavior* by LLR. The major citing article of the cluster is Khan et al. [58] titled *Sustainable tourism policy, destination management and sustainable tourism development: a moderated-mediation model*. The researchers find that sustainable tourism policy, destination management, and destination social responsibility significantly impact sustainable tourism development, with social responsibility partially mediating. In addition, the frequently cited paper of Higgins-Desbiolles et al. [59], argues that the tourism industry's addiction to growth is incompatible with sustainability. The second most cited document is Lee et al. [60]. These authors suggest shifting from tourism's addiction to growth towards a sufficiency approach and propose various strategic solutions.

The 4th largest cluster in light green has 73 members and a silhouette value of 0.892. It is labeled as *collaborative marketing* by LLR. The major citing article here is López et al. [61] *Residents attitude as determinant of tourism sustainability: The case of Trujillo.* They found that perceived benefits have a stronger impact on tourism sustainability than residents' support and that community involvement has a stronger influence on perceived benefits than community attachment. The most cited paper in this cluster is Agyeiwaah et al. [62], with 47 citations. This paper evaluates various indicators for sustainable tourism at the enterprise level, suggesting that simplicity may be the key to progress towards sustainability. The second most cited document is Lee et al. [63]. His paper uses the ambiguous Delphi method and the analytic hierarchy process to identify 141 indicators for sustainable wetland tourism, with stakeholder involvement being compliance with guidelines and reducing environmental impact.

In terms of novelty, the five articles of Buckley R (2012) in Cluster #0, Hall [64] in Cluster #0, Waligo et al. [65] in Cluster #0, Lee et al. [63] in Cluster #3, and Mathew et al. [66] in Cluster #3, are considered the most innovative in the thematic given their sigma score equaling 1.

#### 4.6.2 Cited authors' analysis

**Fig. 6:**
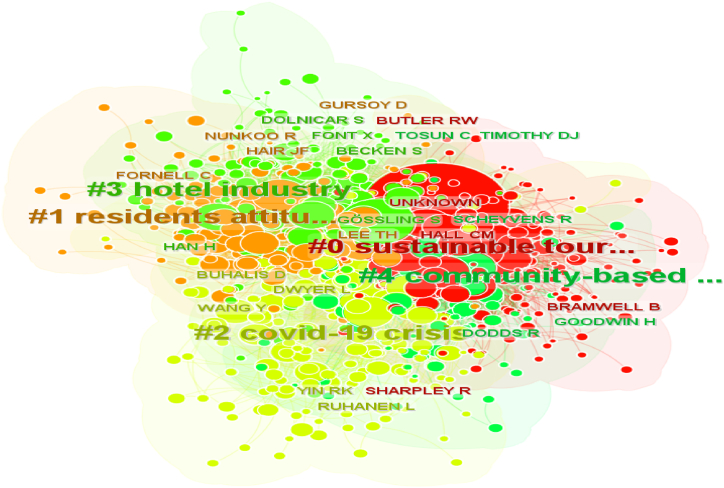
Network of the largest 5 clusters of authors' co-citation.

The network of cited authors is divided into 10 co-citation clusters. The five largest clusters are illustrated in Fig. 6.

The largest cluster (#0) has 232 members and a silhouette value of 0.662. This silhouette score indicates that this cluster is not the most homogenous compared to the other clusters. It is labeled as *sustainable tourism* by both LLRs. The most relevant citer is Cucculelli et al. [67], with the paper entitled: Does sustainability enhance tourism destination competitiveness? Evidence from Italian destinations of excellence”. The study tests a model on destination competitiveness and finds that sustainability positively impacts competitiveness, with a greater impact than other variables, using a sample data set of selected small Italian “destinations of excellence”. The most cited authors are C. Michael Hall with 965 citations and Bill Bramwell with 627 citations. The second largest cluster (#1) has 174 members and a silhouette value of 0.704. It is labeled as *residents' attitude* by LLR. The most relevant citer is Khan et al. (2021), titled Sustainable tourism policy, destination management and sustainable tourism development: a moderated-mediation model. Joseph F. Hair, with 337 citations, and Tsung Hung Lee, with 255 citations, are the most cited authors.

The third largest cluster (#2) has 174 members and a silhouette value of 0.594. It is labeled as *covid-19 crisis by* LLR. The most relevant citer is Huang et al. [68], entitled *Developing a new framework for conceptualizing the emerging sustainable community-based tourism using an extended interval-valued pythagorean fuzzy swara-multimoora*. The most cited authors are Larry Dwyer, with 309 citations and Dimitrios Buhalis, with 307 citations.

The 4th largest cluster (#3) has 137 members and a silhouette value of 0.718. This cluster has the highest homogeneity score and is labeled as *hotel industry* by LLR. The most relevant citer is Bandyopadhyay et al. [69], entitled *Nexus between tourism, hydropower, and co2 emissions in India: fresh insights from ARDL and cumulative Fourier frequency domain causality*. The most cited authors are Stefan Gössling, with 698 citations, and Susanne Becken, with 348 citations.

The top-ranked item by sigma is Richard W. Butler in Cluster #0, with a sigma of 1.00. The second one is Hwansuk Chris Choi in Cluster #5, with a sigma of 1.00. The third is Richard Sharpley in Cluster #0, with a sigma of 1.00. The 4th is Bill Bramwell in Cluster #0, with a sigma of 1.00. The 5th is Cevat Tosun in Cluster #4, with a sigma of 1.00.

#### 4.6.3 Cited journals analysis

**Fig. 7:**
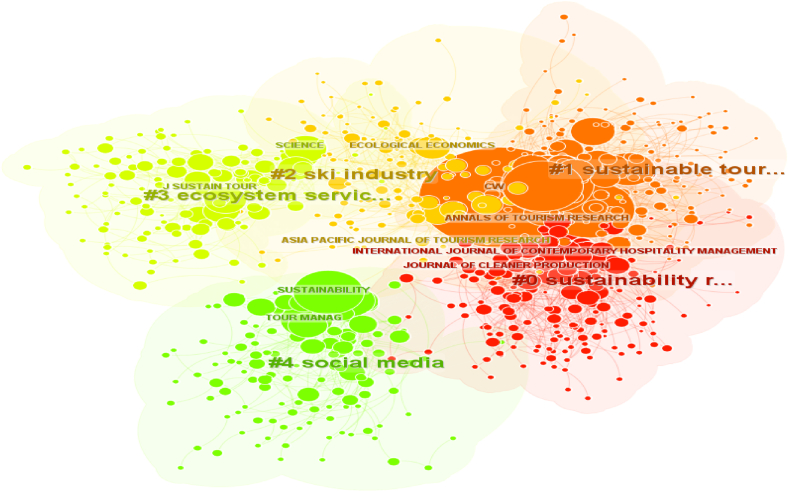
Network of the five largest clusters of the source's co-citation.

Based on the analysis of cited journals and the resulting clusters, the network consists of 17 clusters, with the largest 5 clusters illustrated in Fig. 7.

According to the number of citations, the top-ranked journal is Annals of Tourism Research in Cluster #1, with citation counts of 1942. The second most cited journal is Tourism Management in Cluster #1, with citation counts of 1831. Third is the Journal of Sustainable Tourism in Cluster #1, with a citation count of 1479. The fourth most cited journal is Sustainability in Cluster #4, with citation counts of 1154. Ranked fifth is Current Issues in Tourism in Cluster #1, with citation counts of 1126.

The top-ranked journals by sigma are the Journal of Biological Conservation in Cluster #4. Secondly, Tourism and Sustainability in Cluster #1. Thirdly is Environment and Behavior in Cluster #2, 4th is Sustainable Tourism in Cluster #1, and lastly is Global Environmental Change in Cluster #5.

#### 4.6.4 Keyword co-occurrence

**Fig. 8:**
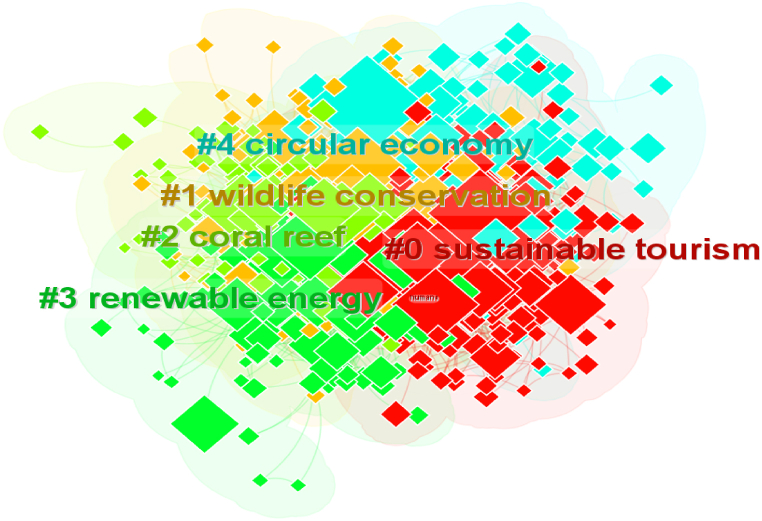
Network of the five largest clusters of the Keyword's co-occurrence.

The keyword co-occurrence network comprises 15 clusters in total. The largest 5 clusters are illustrated in Fig. 8.

In red, the most prominent cluster (#0) has 170 members and a silhouette value of 0.666. It is labeled as *Sustainable Tourism* by LLR. The most cited topics are sustainability, ecotourism and sustainable tourism. The second largest cluster (#1) has 158 members and a silhouette value of 0.592. It is labeled as *wildlife conservation* and includes the most cited topics, namely climate change, conservation and local participation. The third largest cluster (#2) has 151 members and a silhouette value of 0.779. It is labeled as a *coral reef* by LLR, and the most cited topics are environmental protection, environmental sustainability and protected area.

The 4th largest cluster (#3) has 115 members and a silhouette value of 0.604. It is labeled as *renewable energy* by LLR. The most relevant topics are economic development, economic growth, and ecology, and this cluster is related to economic sustainability. The 5th largest cluster (#4) has 105 members and a silhouette value of 0.64. It is labeled as a *circular economy* by LLR.

##### 4.6.4.1 Conceptual map of cluster (#0) and sustainable tourism

These conceptual maps are built from the co-occurrence networks of each cluster. The hierarchy of the themes is based on the degree of centrality that measures the number of connections a node has with other nodes based on cooccurrences. Nodes with higher degrees of centrality indicate greater connectivity and influences within the network, reflecting their significance in understanding relationships and patterns between elements or entities [70].

**Fig. 9:**
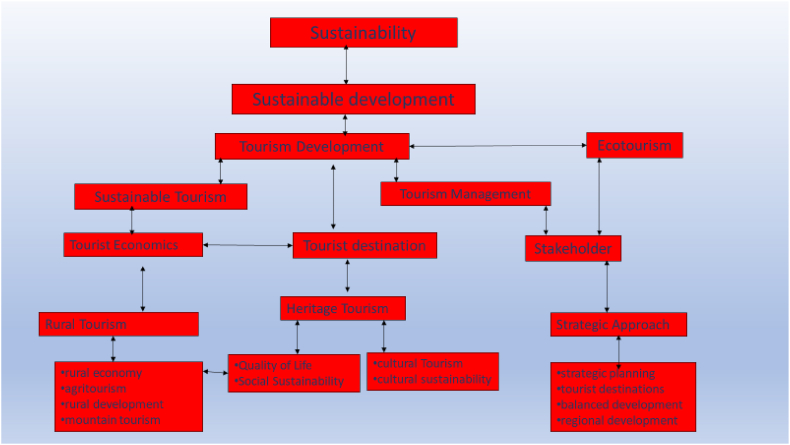
Conceptual map of the sustainable tourism cluster.

Fig. 9 highlights the need for sustainable tourism practices that consider all stakeholders' interests and address destination management challenges. The topics related to sustainable tourism include “sustainable development,” “sustainable tourism,” “sustainable tourism development,” and “green tourism.” These topics discuss the importance of sustainable practices in the tourism industry and the need to balance economic, social, and environmental factors to ensure long-term viability.

Looking at Table 7, this cluster firstly deals with the topic of **sustainable tourism**. Zarokosta et al. [48] emphasize the importance of utilizing local resources, entrepreneurship, and stakeholder collaboration for sustainable tourism development in rural areas. Stone et al. [71] examine the challenges in conceiving, designing, and implementing community-based tourism in Botswana, including issues related to community definition, business acumen, and income distribution. García-Melón et al. [72] propose a new multicriteria approach to decision-making for sustainable tourism in coastal national parks in Venezuela. Pereira et al. [47] explore the relationship between consumers' sustainable purchasing practices and their related cognitions, evaluations, and beliefs when considering purchases of tourist products or general goods.

**Table 7:** Cluster (#0) related topics and references.

Basic topics	Related topics	Citing references
Tourism Development	Ecotourism, Sustainable Tourism, Tourist Destination, Tourism Industry, Tourism Management, Tourism Market, Sustainable Tourism Development, Tourism Sector, Tourist Behavior, Tourist Attraction, Tourist Destinations, Tourism Sustainability, Tourism Destinations, Marketing, Innovation, Competitiveness	[[73], [74], [75], [76], [77]]
Stakeholder	Stakeholder Engagement, Stakeholder Management, Stakeholder Analysis, Stakeholder Mapping	[[78], [79], [80], [81]]
Perception	Consumer Perception, Tourist Perception, Destination Image, Destination Branding	[[82], [83], [84], [85]]
Tourism Economics	Tourism Demand, Tourism Supply, Tourism Multiplier, Tourism Impact, Tourism Contribution to GDP	[[51], [86], [87], [88]]
Corporate Social Responsibility	Social Responsibility in Tourism, Ethical Tourism, Sustainable Business Practices, Responsible Tourism	[[50], [89], [90]]
Strategic Approach	Tourism Strategy, Destination Strategy, Marketing Strategy, Innovation Strategy, Competitive Strategy	[49,91]

The second topic is **cultural sustainability**, which focuses on preserving and promoting cultural heritage while supporting local development. Ghirardello et al. [92] explore the relationship between tourism and intangible cultural heritage and proposes ways to promote sustainable governance that considers cultural heritage preservation while supporting local tourism development. Gonçalves et al. [93] highlights the importance of using digital technologies to promote cultural heritage and engage visitors in meaningful experiences. Lonardi [94] highlights the importance of cultural sustainability in maintaining traditional languages and practices. Hayajneh et al. [95] detail how UNESCO, the Jordanian government, and local communities work together to develop a knowledge-based economy, protect natural and cultural heritage, promote intercultural dialogue, and institutionalize cultural development.

The last topic is that of **social sustainability**. McCombes et al. [96] emphasize the need to shift the discourse on the social impacts of tourism from descriptive critiques to practical solutions. Despite various tourism initiatives in indigenous areas, Brandão et al. [97] discuss the lack of public tourism policies and regulations for indigenous tourism in Brazil. Madeira et al. [98] argue that Airbnb houses should be considered micro-enterprises with features that can help achieve social sustainability, including the potential to involve poor local people in the tourism industry and expand the benefits of tourism through partnerships with other service providers. Stevenson [99] examines the social capital generated by local festivals in an emerging destination. While these festivals increase social capital in the area, they also exacerbate existing inequalities within the community and create tensions between social capital and social sustainability.

##### 4.6.4.2 Conceptual map of cluster (#1)

**Fig. 10:**
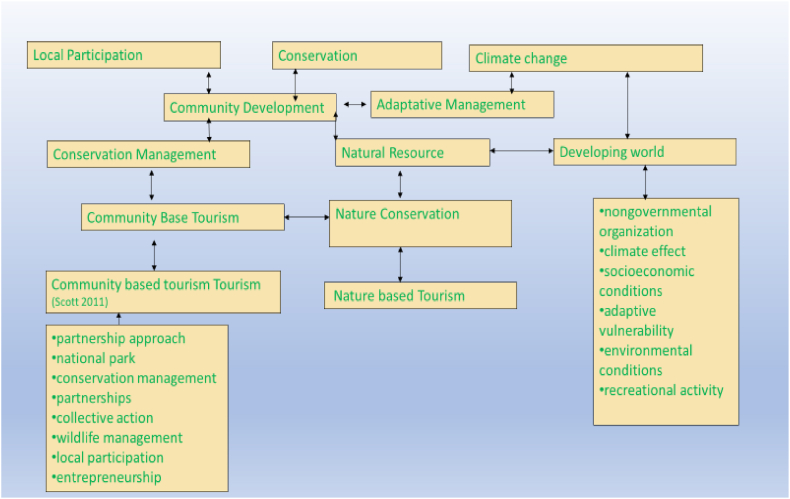
Cluster (#1) conceptual map.

The conceptual map of the conservation cluster is illustrated in Fig. 10. The hierarchy of the themes is based on the degree of centrality.

This cluster illustrates the interconnectedness of various topics related to sustainable tourism. The main theme is “Sustainable Tourism,” which serves as the central node. Table 8 illustrates the topics covered by the cluster and the references dealing with this theme.

**Table 8:** Cluster (#1) related topics and references.

Basic topics	Related topics	Citing references
Sustainable Tourism	Conservation Management, Climate Change, National Park	[[100], [101], [102], [103]] [104]
Conservation management	Nature Conservation	[[105], [106], [107]]
Climate Change	Climate Effect, Environmental Change, Developing World.	[[108], [109], [110]]
National Park	Marine Park, Nature Society Relations	[[46], [111], [112]]
Recreational Activity	Nature-based Tourism, Outdoor Recreation	[[113], [114], [115]]
Economic Activity	Entrepreneurship, Performance Assessment	[[116], [117], [118]]
Local Participation	Community Development, Public Participation	[[45], [119], [120]]
Wildlife Management	Conservation Policies, Species Conservation	[[44], [121], [122]]

Basic Topic	Second-Order Topics
Sustainable Tourism	Conservation Management, Climate Change, National Park
Conservation Management	Wildlife Management, Nature Conservation
Climate Change	Climate Effect, Environmental Change
National Park	Marine Park, Nature Society Relations
Recreational Activity	Nature-based Tourism, Outdoor Recreation
Adaptive Management	Resilience, Vulnerability
Economic Activity	Entrepreneurship, Tourism Economics, Performance Assessment
Local Participation	Community Development, Public Participation
Entrepreneurship	Small Business Development, Social Entrepreneurship
Wildlife Management	Conservation Policies, Species Conservation

From Table 8, the cluster deals with several topics relating to **sustainable tourism in the developing world.** Here, [123] examine the complexities of pro-poor tourism in developing nations, highlighting the ideological divisions among stakeholders and the challenges of achieving both pro-poor and sustainable development objectives within a neoliberal market economy [124]. emphasizes the need for aligning sustainable tourism management policies with national energy and environmental policies to optimize economic gains from tourism while mitigating detrimental environmental effects [125]. examines the relationship between fisheries biodiversity and the sustainability of the tourism sector in Mauritius and emphasize the significance of conserving biodiversity, specifically in the fisheries sector, to maintain economic advancement in the country [126]. explore the influence of knowledge-intensive intermediaries, exemplified by the South African organization ‘Fair Trade in Tourism,’ in shaping global standard-setting processes and highlights the need for a dynamic and context-specific approach to integrating the perspectives of actors from developing countries in sustainable development efforts.

##### 4.6.4.3 Conceptual map of cluster (#2)

**Fig. 11:**
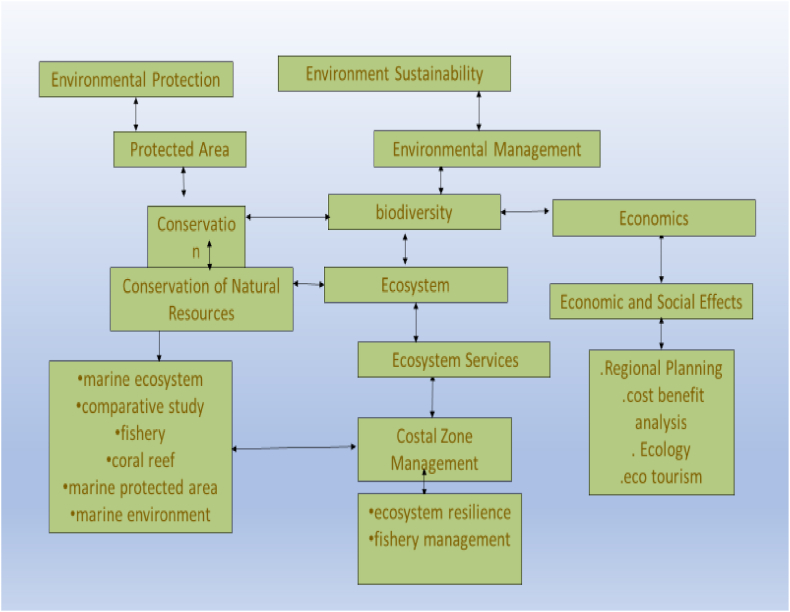
Conceptual map of the environment sustainability cluster.

Fig. 11 outlines the conceptual map for the environment sustainability cluster.

This cluster revolves around the theme of environmental management, which involves the conservation, protection, and sustainable utilization of natural resources. It recognizes the significant impact of human activities, particularly in the agricultural and coastal zones, and aims to integrate environmental concerns into economic decision-making processes. Table 9 provides a graphical representation of the cluster's encompassed topics and the corresponding references pertaining to this thematic area.

**Table 9:** Cluster (#2) related topics and references.

Basic topics	Related topics	Citing references
Environmental Management	Environmental Impact, Biodiversity Land Use, Protected Areas, Wildlife Conservation, Forest Management Sustainable Agriculture	[[127], [128], [129]]
Environmental Protection	Pollution Control, Waste management, Environmental Legislation, Conservation of Natural Resources, Environmental Monitoring	[[43], [130], [131]]
Agriculture	Sustainable Agriculture, Crop Production, Soil Management Irrigation, Livestock Farming Agroforestry	[[132], [133], [134]]
Environmental Sustainability	Sustainable Tourism, Renewable Energy, Green Building, Water Conservation, Circular Economy, Carbon Footprint, Ecosystem Restoration	[[135], [136], [137]]

Dealing with **environmental sustainability** in this cluster, Alcay et al. [138] suggests that socio-economic factors such as per capita income, environmental spending, and education level contribute to this variation. Li et al. [139] finds that cross-cultural exchanges between tourists and locals can contribute to social value creation and environmental sustainability. Sáez-Fernández et al. [140] examine the impact of tourism seasonality on the efficiency of hotels in Spain and finds that hotels that remain open during the off-season are more efficient in using inputs and overall performance compared to those that close down. Pereira et al. [141] explore the environmental and social sustainability practices luxury hotels adopt and their perceived benefits and results.

##### 4.6.4.4 Conceptual map of cluster (#3)

**Fig. 12:**
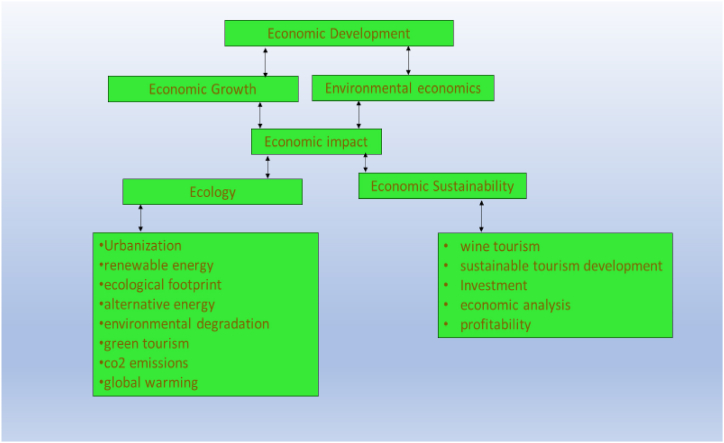
Conceptual map of the economic sustainability cluster.

Fig. 12 outlines the conceptual map for the economic sustainability cluster.

The cluster of economic sustainability encompasses topics such as environmental economics, economic development, economic analysis, investment, and economic growth. These topics explore the connection between economics and sustainability by examining the economic impacts of environmental issues, the growth and improvement of economic systems, and the allocation of resources for future benefits. Table 10 presents the topics included within the cluster, along with the references that address the subject matter of this specific thematic area.

**Table 10:** Cluster (#3) related topics and references.

Basic topics	Related topics	Citing references
Ecology	Environmental Degradation Carbon Dioxide Emissions, Pollution	[[142], [143], [144]]
Environmental Economics	Economic Development, Economic Analysis, Economic Impact	[[145], [146], [147]]
Carbon Dioxide	Carbon Emission, Carbon Footprint, Global Warming	[[148], [149], [150]]
Urbanization	Infrastructure, Affordable Housing, Transportation	[151,152]
Economic Growth	Gross Domestic Product, Industry, Employment	[153,154]
Environmental Degradation	Environmental Impacts, Sustainable Practices	[155,156]

The studies in this cluster focus on the relationship between a sustainable economy and tourism, emphasizing the importance of community involvement, green tourism, and sustainable livelihoods. Rahman et al. [157] examine the impact of community participation on sustainability in marine protected areas in Malaysia, highlighting the importance of community involvement in achieving sustainable development. Kadriu [158] emphasizes the potential of Green Tourism to create new jobs and contribute to the overall stability of the economy. Blekesaune et al. [159] analyze the visitors who engage in various activities and services offered by Norwegian farms and provides insights into the characteristics of potential visitors in the domestic tourism market. Yfantidou et al. [160] discuss the concept of ‘green tourism’ and examine the potential for sustainable tourism development in Greece, specifically in sport tourism, while considering the changing values of tourists concerning environmental issues. Chen et al. [161] utilize group model building and system dynamics to manage Jiading Wetland amidst rapid economic growth and population increase to ensure tourism sustainability. Su et al. [162] examine the impact of tourism and resettlement on the livelihoods of a rural community in China and whether tourism-related strategies contribute to community livelihood sustainability, highlighting the need for measures to diversify livelihood options and mitigate potential challenges for the affected community. Uzzo [163] examines the criticisms raised on the long-term impacts of resort operations on local and regional economies, environment, and culture.

## 5 Discussion

Tourism has always been a significant contributor to economic growth and development. Unfortunately, the Covid-19 pandemic halted this sector to a total standstill in 2020 and 2021, with travel and other rigid restrictions being imposed to reduce the spread of the virus. As economies are opening and recovering, the tourism sector is slowly recuperating. As global warming leads to more changes in weather patterns, sustainability and sustainable tourism has been a serious topic of discussion in recent years. Although tourism is not the main cause of global warming, it is undoubtedly one of the contributors [164]. Through overconsumption and inadequate management, tourism pressures the environment and its resources. Increased usage of transport methods increases gas emissions and adds more consumables and disposable waste. This makes it vital to continuously conduct new and updated research on sustainable tourism.

As this study's main objective was to showcase the research in tourism sustainability from 1990 to 2021, it set out to answer three important research questions. These were (a) to identify the most fruitful authors, sources, and affiliations within the research domain of tourism sustainability, (b) to determine the intellectual structure through identifying and analyzing to most important co-citation clusters in tourism sustainability, and (c) to design and develop a research agenda for future work on this important topic. This study adopted two methods. Firstly, the descriptive analysis was done using R4.1.2, and secondly, co-citation clusters for authors, documents, sources and keywords were identified using the CiteSpace 6.0 R1 software [31].

This analysis revealed interesting and valuable results from the literature related to tourism sustainability. As seen in Fig. 2, the theme of tourism sustainability has become more popular over time. Since 1998 a steady increase in the number of publications has been seen. Since 2013, this trend has increased significantly, with more than 250 publications that year rising to close to 1000 in 2021. The most productive journal was *Sustainability,* and although this journal does not exclusively focus on tourism topics, a total of 745 papers have been published on this topic. Similarly, it also produced the highest growth and considering the journal only published its first article on this topic in 2013, this achievement is rather significant. In addition, the journal occupies a good position regarding citations.

From the author dominance analysis Gössling S, Weaver D and Hall C produced the most single-authored papers highlighting their expertise in this field. Hall C was also among the most productive authors, with Font X as the most productive. These authors' research strongly focuses on transportation, mobility, tourism geography, sustainability, regional development, global environmental change, Sustainable Development Goals and tourism marketing. The top 10 most productive authors are affiliated with the University of Surrey (UK), University of Canterbury (NZ), Linnaeus University (Sweden); Oulu University (Finland); Shandong University (China), The Hong Kong Polytechnic University, Griffith University (Australia), Lund University (Sweden), University of Queensland (Australia), Toronto Metropolitan University (Canada) and Texas A&M University (USA). Interestingly, more than half of the leading authors are from Asian Pacific countries, and no representation is seen from developing countries.

In order to determine which topics received specific attention from the scientific community over certain periods, the citation bust method was used [165]. The older bursts, tourism development (1996–2003), tourism management (1999–2005) and sustainability (1999–2003), already highlighted the importance of the topic of sustainable tourism. Several bursts were noticed that focused on specific countries or regions such as Europe, Australia, Eurasia, Asia, the Eastern hemisphere, Africa, Australasia and North America. This emphasizes the importance and interest of research at national and regional levels. Several studies during these bursts also focused on comparisons between areas. Since 2008 the busts started focusing explicitly on sustainability topics such as conservation management (2008–2013), conservation of natural resources (2011–2018), nature-based tourism (2012–2018), ecology (2012–2018) and over-tourism (2019–2021).

From the co-citation analysis, several clusters emerged. From the paper co-citation analysis, the emerging clusters identified were regarding the small island states, conceptualizing tourism transport local development and theoretical divide. The largest cluster focused on the small island states, also more formally known as Small Island Developing States (SIDS), with a member tally of 142. Research on this topic has been increasing as SIDS is considered vulnerable to over-tourism during peak seasons and low growth and development due to their smallness and remoteness. Scheyvens and Momsen (2008) state that the perception that SIDS are always vulnerable to over-tourism, consisting of mostly unskilled people with limited resources and economically dependent, may undermine some of these communities.

The authors argue that research should also highlight some of these island states' positive aspects. These include demonstrating strong social dimensions of sustainability and showing resilience and adaptability. The second largest cluster also addresses an important topic concerning tourism transportation. This cluster had 86 members and a silhouette of 0.986. Several important research questions on this topic have been raised in the past. Hall ([39]:181) points out that “the actual and symbolic representations of inequality and differentiation expressed in leisure and tourism mobility which have significance for members of host communities visited, transport and land-use planning in host areas, tourists and the tourism industry”. He further opines that transport has the potential to act as a gatekeeper to the cultural side of tourism and can either constrain or encourage interaction by the host country. In addition, the role of tourist mobility is crucial for local growth and development and can affect issues such as inequality. More recently, Peeters et al. [166] emphasized the importance of the impact of tourism transportation on climate change. Although the topic of tourism transportation has often been investigated, less attention has been given to sustainable tourism transport.

The co-citation analysis of the top five authors revealed the following cluster themes: tourism, with 106 members, and international resort industry, with 78 members and a recent development, adaptive change and land use choices. The importance of tourism has been highlighted, and the industry is recovering from the impact of the recent pandemic. The topic of international resorts has been investigated in many research studies. The estimated market size was $1.06 trillion in 2021 (down from $1.52 trillion in 2019) [167]. This makes the research of international resorts a vital topic of discussion. Responsible management of international resorts, which are often in close proximity to ecologically sensitive areas, is crucial. As more focus has been placed on sustainability, many of these resorts also came into the spotlight, and more emphasis has been placed on sustainable practices [168]. This opened the door for additional and updated research on the topic of international resorts and sustainability [169].

The research found that incorporating good management practices through sustainable initiatives can positively influence business operations [168]. Many tourists are aware of sustainability issues and would be more accepting of resorts following sustainable practices. Considering the co-citation clusters of sources, the top five emerging clusters were regional perspective (160 members), sustaining tourism employment (118 members), the international resort industry, scale agriculture and nascent conservation. Research on tourism's contribution to regional and local development is crucial. Many local communities rely on tourism as a means to survive. The topic of this cluster links well to the next largest cluster, as sustainable tourism employment is also a topic of importance. The exploitation of workers is often seen in the tourism industry. Tosun et al. [170] stress the importance of tourism employment to alleviate regional inequality and poverty. Results indicate the lack of correlation between tourism growth and reduced unemployment and that more should be done in tourist regions to improve positive spillovers to workers. One of the challenges they point out is that tourism employment is seasonal in many regions, leading to unsustainable and fluctuating benefits for these workers. Many workers are low-skilled and rely heavily on their involvement in tourism-related jobs.

Finally, the top 10 cited publications were identified. The oldest was published in 1993 with 57 citations (ranked 6th). This editorial focused on the evolving approach of sustainable tourism. Some basic principles recommended included that holistic planning and strategy-making are crucial, ecological processes, human heritage and biodiversity should be preserved, and that development should be done in a way as to protect and ensure sustained biodiversity and heritage [171]. The top-cited paper by Liu [41] received 96 citations over a period of 19 years. This paper critiques some shortcomings in research conducted on sustainable tourism. More specifically, it explores overlooked and under-researched (at that time) topics such as tourism demand, resources, intra-generational equity, tourism as a mode to promote socio-cultural progress, how to measure sustainability and different forms of sustainable development. The author suggests that tourism research should be transformed into a more scientific level, improved systems perspective and an interdisciplinary approach. In the last two decades, this research approach has evolved and addressed the recommendation to a certain level, especially regarding the use of a more interdisciplinary approach. The most recent publication was that of Buckley in 2012, with 85 citations. This paper reviewed around 5000 publications on the topic of social and environmental impacts of tourism and found that very few access the tourism sector in terms of global research in sustainable development. He further opined that the industry is far from sustainable. Other important observations were that political approaches to avoid environmental restrictions and gain access to public natural resources were still widely used.

## 6 Conclusion

This study aimed to conduct a comprehensive bibliometric and systematic review of tourism sustainability. A total of 6326 publications were retrieved from the Scopus database. The main results indicated a remarkable evolution of tourism sustainability research involving authors, sources, and publications on this subject. Several associations and nations made significant contributions to this theme. Based on the analysis and findings we used in the analysis and findings; we list several implications.

### 6.1 Implications and direction for future research agenda

The use of bibliometric reviews has increased in recent years and is useful for obtaining trustworthy indicators related to the progression in a specific research field. Our study revealed valuable insights regarding the top authors, citation bursts and co-citation clusters. These findings have several theoretical and managerial implications that need to be considered for future research. Regarding the theoretical implications, the findings of this study suggest that although tourism research has been increasing exponentially over the few decades, the theme of sustainability and the importance of the SDG's lacking throughout the research. It would seem that sustainability is only being touched on and not comprehensively researched in terms of its role in tourism and how the industry could move towards a more sustainable future. This leads to further managerial implications as the practicality of integrating sustainable practices within the tourism industry remains vague. The tourism industry heavily relies on a region'segion's social, cultural, and environmental. Without this, policies and destination management in tourism-reliant regions would most likely not be constructive. Based on the findings, we propose several future research streams that can enrich the topic of sustainable tourism.

#### 6.1.1 Sustainable Development Goals

The positive contributions of tourism to employment and economic growth are not enough. Recent attention to sustainability, specifically relating to the United Nations Sustainable Development Goals (SDGs), is noteworthy. The overall objective of the 17 SDGs is to reduce poverty and inequality and reduce the effects of climate change – all of which could be positively affected by sustainable tourism practices. Concerning tourism, some goals particularly stand out. Tourism has been particularly included as an objective in Goals 8, 12 and 14. These focus on sustainable and inclusive growth, production, consumption, and the use of ocean marine resources in a sustainable manner [172]. One noticeable shortcoming of the findings is the lack of mention of the SGDs. This could be due to them only being adopted in September 2015. However, even papers included after this date lacked the inclusion of these important goals [173]. Future research on this important topic is crucial.

#### 6.1.2 Carrying capacity

The geographical concept of carrying capacity refers to the maximum capacity of something which is needed to sustain something else [174]. Within tourism, there are three types of carrying capacity indicators; 1) physical refers to the maximum amount of people who can occupy a space for tourism uses, 2) perceptual refers to the level reached when residents in a specific area prefer not to have fewer tourists, regardless of the economic benefit because they are destroying the environment, causing damage to the local culture or crowding the local community out of local activities [175] and 3) environmental capacity which affects the ecological environment in such a way that it is worse off and compromised than before the tourist development. A lack of this research focus was noticed, and future research on this topic is not only important but can be interesting and useful.

#### 6.1.3 Desirable tourism transport futures

Peeters et al. [166] emphasize the importance of moving tourism transport into a sustainable emissions path. This trend may be more noticeable in developed countries, but emerging economies do not always have the resources to focus on lower emissions. Although some mention of this was made in one of the clusters, there is still much room for research on this topic. Specific attention should be made to tourism transport in developed and developing countries as there are many disparities between development levels. Also, pertinent links should be made to the UN SDGs.

#### 6.1.4 Over-tourism and the role of tourism in protected areas

The challenge of managing tourism sustainably for residents and tourists has been recognized for the past two decades. However, the hypothesis developed around tourism has largely stated that tourism is mainly “good” and often sugar-coated with the words sustainable and sustainability liberally included to reassure negatively impacted communities and critics [176]. There has been a shift in the local community's perceptions of tourism in recent years; a tipping point has been reached in many destinations. Mass tourism has become a local political issue, sometimes resulting in unrest and protests. With the tourism sector expected to grow to 1.8 billion people by 2030 [177], attention should be paid to managing tourism and limiting over-tourism. Although research on this topic is available, this study lacked identifying this aspect in the results. Considering the importance of this topic and its link to sustainable tourism, future research is needed. New research should not focus on the causes of the occurrence but rather on the destinations that deal with the problems progressively, the associated implications of the occurrence, and possible solutions.

### 6.2 Limitations

Although this study brought new insights into the topic of sustainable tourism, it is not without some limitations, as any research, which leads to substantial opportunities, directions, and recommendations for future research. The first limitation is linked to the bibliometric approaches utilized in this paper. The current paper employed a comprehensive bibliometric approach, including the analysis of the domain or the key trends in this area of research as well as the knowledge structures of tourism sustainability research during the given period. As a result, future studies in this area of research are recommended to review and systematically analyze and map tourism sustainability research methods, procedures and techniques of data gathering, and data analysis approaches and methods. The second constraint is that the current study focused on peer-reviewed articles written in English and published in Scopus-indexed journals. Future studies are proposed to retrieve and analyze data from other databases, such as Web of Sciences, Google Scholar, etc. This will help gather a wider range of publications on the theme. Moreover, future research can conduct a bibliometric study to examine editorials, research notes, book reviews, book chapters, and conference proceedings. These publications could include potential and remarkable early-stage notions, trends, and ideas that have yet to appear in academic journals [178]. This aids in gaining a better understanding and insight into the major trends, concerns, and knowledge structures concerning tourism sustainability. In addition, further research is recommended to focus on tourism sustainability publications published in other languages. Indeed, this could provide a valuable and in-depth understanding of this area of research.

## Production notes

### Author contribution statement

All authors listed have significantly contributed to the development and the writing of this article.

### Data availability statement

Data included in article/supp. material/referenced in article.

### Additional information

No additional information is available for this paper.

## Declaration of competing interest

The authors declare that they have no known competing financial interests or personal relationships that could have appeared to influence the work reported in this paper.
